# Unilateral Cysticercosis of the Parotid Gland: Case Report, Diagnosis, and Clinical Management

**DOI:** 10.1155/2021/9999441

**Published:** 2021-07-31

**Authors:** Zachary Elwell, Shethal Bearelly, Khalid Aboul-Nasr, Jonathan Lara

**Affiliations:** ^1^University of Arizona College of Medicine, Department of Otolaryngology-Head and Neck Surgery, Tucson, AZ, USA; ^2^Tucson Pathology Associates, Tucson, AZ, USA; ^3^Sonoran Ear Nose Throat, Tucson, AZ, USA

## Abstract

Cysticercosis is a systemic parasitic infection caused by the establishment of the larval form of the parasitic cestode, *Taenia solium*. Cysticercosis is acquired via the fecal-oral route and is prevalent in low- and middle-income countries (LMICs). Patients typically manifest with skeletal muscle, subcutaneous, or central nervous system involvement. Though there are reports of oral mucosa involvement, solitary involvement of the parotid gland is rare. This is a rare case of a 57-year-old man diagnosed with parotid cysticercosis by imaging and FNA. He was successfully treated by anthelminthic therapy and needle aspiration. The patient has been seen back several times. The cyst is not palpable, and he is satisfied. Parotid cysticercosis should be considered in the differential of a parotid mass in patients who have traveled to endemic regions. Though prior reports have indicated the importance of surgical excision, this patient was treated medically.

## 1. Introduction

Cysticercosis is a parasitic disease caused by the larval stage of the cestode, *T. solium*. Cysticercosis is endemic to many LMICs that lack proper sanitation, resulting in environmental fecal contamination of both food products and water [1]. Regional locations include nations in Africa, Asia, and Latin America. Thus, patients who travel to these regions are at an increased risk of acquiring cysticercosis.

Symptom presentation of cysticercosis is variable and is dependent on the location and number of cysts (cysticerci) involved [2]. Tissues commonly affected include skeletal and cardiac muscle, skin and subcutaneous tissue, the CNS, and rarely, the oral mucosa [1–3]. In most locations, cysticerci spontaneously degenerate. However, establishment to the CNS can result in neurocysticercosis (NCC) which may be associated with serious neurologic manifestations [1, 2, 4]. According to Catney et al., in 2014, nearly 2% of emergency department visits for seizures were due to NCC. Thus, cysticercosis has been increasingly recognized as a cause of severe and preventable neurologic disease in the U.S [1].

Traditionally, cases of cysticercosis are diagnosed by imaging and serological methods. Histopathological examination of excised lesions can be performed after the time of surgery [3–5]. The most recent cases of cysticercosis were diagnosed through the use of preoperative fine-needle aspiration cytology (FNAC) and allow for a trial of pharmacologic intervention with anthelminthic therapy–including praziquantel–before surgical intervention [6, 7]. In the case that follows, we present a patient with a rare presentation of cysticercosis of the parotid gland diagnosed by FNA and successfully treated with praziquantel.

## 2. Case Presentation

A 57-year-old man presented with a chief complaint of right facial swelling after traveling to South Korea. Physical exam demonstrated a palpable right parotid mass. The patient had no other symptoms and denied dysphagia, pain, swelling, or difficulty opening their jaw.

**Figure 1:**
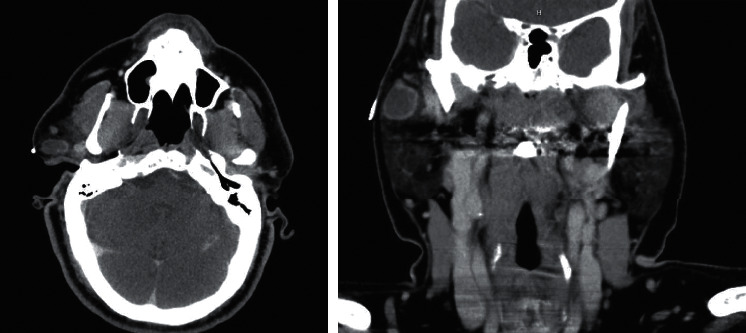
Axial and coronal plane CT neck with contrast demonstrating a 2 cm rim-enhancing lesion within the right parotid gland corresponding to the cestode infection.

CT scan of the neck with contrast demonstrated a 2.1 × 1.0 × 2.0 cm ovoid low-density rim-enhancing lesion in the superficial lobe of the right parotid gland (Figure 1).

**Figure 2:**
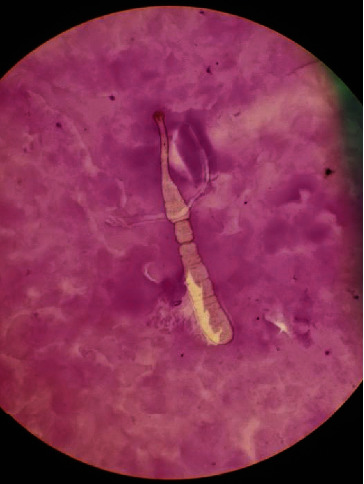
Microscopic examination at 40x magnification demonstrated the neck, scolex, and partial strobila of the cestode in a background of secretory debris.

FNA of the right parotid and right lymph node demonstrated benign cyst contents from the parotid, from the right lymph node, and a polymorphous lymphoid population consistent with lymph node sampling. There was no cytologic evidence of malignancy. However, a single degenerated structure suspicious for the neck, scolex, and partial strobila of the cestode was identified (Figure 2). The slides were sent to the Centers of Disease Control (CDC), and it was confirmed to be a cestode. Further classification of the cestode was not possible due to the degenerated nature of the organism. Given these findings, the patient was prescribed two doses of praziquantel 600 mg over a 10-day period for a total of 10 mg/kg and advised to follow-up in 3 months.

**Figure 3:**
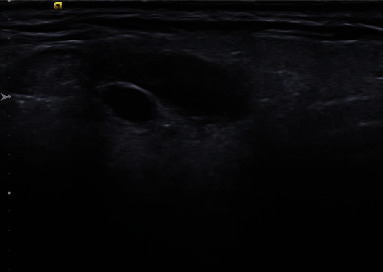
Ultrasound of the right parotid revealing residual hypoechoic/cystic lesion which was subsequently aspirated.

The patient returned and noted some residual right facial swelling. The mass was nontender, and the patient denied dysphagia or pain with chewing. Of note, the patient had not traveled and had no further infectious exposures. An ultrasound was performed and revealed a residual 1.9 × 0.9 × 0.8 cm hypoechoic lesion within the right parotid gland (Figure 3). A repeat FNA was performed and yielded another 2 cc of clear fluid with only secretory debris without evidence of a cestode. At this time, further workup was offered including potential surgical excision, but the patient wished to defer this due to continued travel plans.

The patient returned 6 months following the initial diagnosis, and his swelling and symptoms were much improved. At the time of publication, he continues to do well without any further treatment.

## 3. Discussion

*T. solium* is a cestode parasite (tapeworm) that consists of a head (scolex) with suckers and hooklets, a neck, and numerous flat segments that contain both male and female reproductive structures [2]. *T. solium* causes cysticercosis infections secondary to the development of larvae after the ingestion of tapeworm eggs. Cestodes have a complex life cycle that requires two mammalian hosts: a pig intermediate host, which supports the nonreproductive form of the parasite, and a human definitive host, which supports the reproductive form [1, 2]. *T. solium* cysticerci establish in tissues where they degenerate after a certain period and cause a local inflammatory response. When intermediate hosts (pigs or humans) ingest eggs in food or water contaminated with feces, the egg hatches and the oncosphere penetrates the gut wall [1, 2]. The oncospheres spread hematogenously, allowing the parasite to reach many organs. This pattern of dissemination and subsequent degeneration of the oncospheres gives rise to the clinical symptoms of cysticercosis [1, 2, 4, 8]. Symptoms usually present within the CNS, with the most serious manifestations resulting in convulsions, increased intracranial pressure, and other neurologic disturbances [1, 2, 4, 8]. Intact cysticerci themselves typically avoid host immune detection. However, when they die and degenerate, an inflammatory response develops resulting in local inflammation [2].

Cysticercosis of the oral mucosa is rare, and according to a case report by Sharma and Kaur, only 38 cases of oral cysticercosis have been published in the literature as of 2017 [3]. Cases of parotid cysticercosis are even less common with just 5 cases published between 2007 and 2017 [3–7]. Among these 5 cases, the most consistent clinical characterization of parotid cysticercosis is the presence of a solitary parotid mass that is well-circumscribed, mobile, nontender, and asymptomatic [3–7]. A notable exception is a case presented by Chakraborty et al. in which a patient was initially diagnosed with NCC which subsequently disseminated to the left parotid gland, left rectus muscle, and skin overlying the right lateral malleolus [4]. This case highlights the importance of considering cysticercosis in the differential diagnosis of a solitary parotid mass due to the establishment of cysticerci in a variety of different tissues.

The diagnosis of parotid cysticercosis should be suspected in patients with a solitary, well-circumscribed, mobile, nontender parotid mass. The diagnosis is established based on clinical manifestations, imaging findings, cytopathological analysis, and epidemiologic exposure [3–8]. The method for establishing a diagnosis of parotid cysticercosis has changed over the past decade. A case report by Seith et al. utilized ultrasonography, MRI, and cysticercosis immunoblot serology to establish a diagnosis that was confirmed with surgical excision of the parotid nodule and postoperative histopathologic examination [5]. Similarly, Chakraborty et al. performed an MRI of the parotid which was suggestive of a cyst before excision biopsy and postoperative histopathological examination for confirmation of parotid cysticercosis [4]. Subsequent studies have demonstrated equal efficacy in establishing a diagnosis of parotid cysticercosis without the need for surgical intervention by utilizing US, MRI, and FNAC with May-Grunwald-Giemsa (MGG) staining [3, 6]. Typical US findings demonstrate cystic lesions with echoic foci which, in some cases, are suggestive of a scolex [3, 5]. Typical MRI findings demonstrate hypointense foci involving a cyst, suggestive of a scolex [4, 5]. Typical FNAC and MGG findings demonstrate lymphocytes, histiocytes, and occasional neutrophils against a granular background with the absence of eosinophils, giant cells, granuloma, or atypical cells [3, 6]. In some cases, evidence of a scolex was visible. Notably, none of the 5 cases published between 2007 and 2017 documented the use of CT imaging in the diagnosis of parotid cysticercosis. The success of FNAC with MGG analysis has allowed practitioners to utilize trials of medical intervention with anthelminthic therapy before surgical intervention. This has averted the need for surgery in at least 2 cases as documented by Goyal et al. and Veena et al. [6, 7].

Treatment of cysticercosis was successful in all previously documented cases and has become less invasive in recent reports [3–7]. Earlier presentations were managed with excision of the parotid lesion and postoperative histopathological examination of the tissue for diagnostic confirmation [3, 5]. This method has been replaced by less invasive modalities including US, CT, MRI, and FNAC [3–7]. These modalities can identify cystic structures with eccentric nodules that represent a scolex which, when identified, is pathognomonic of cysticercosis [5]. Seith et al. described the utility of enzyme-linked immunosorbent assay (ELISA) or enzyme-linked immunoelectrotransfer blot (ETIB) in cerebrospinal fluid serologic investigations for patients with suspected NCC and reported EITB to be more sensitive and specific than ELISA [5]. They further recommend MRI of the brain is obtained in all patients diagnosed with cysticercosis to rule out associated NCC. Surgical excision of accessible solitary lesions is a viable treatment method [5]. However, there has been increasing documentation of successful treatment with anthelminthic therapy, a trial of which may be warranted in some cases before surgical intervention [4, 6, 7].

## 4. Conclusion

This is a rare case of a 57-year-old man diagnosed with parotid cysticercosis by imaging and FNA. He was successfully treated by needle aspiration and anthelminthic therapy. Parotid cysticercosis should be considered in the differential of a parotid mass in patients who have traveled to endemic regions. Though prior reports have indicated the importance of surgical excision, this patient was treated medically.

## Data Availability

The data used to support the findings of this study are available from the corresponding author upon request.
